# Interactions between attained proficiency and length of exposure to lexical attrition of English as a second language

**DOI:** 10.3389/fpsyg.2025.1586722

**Published:** 2025-09-17

**Authors:** Hui Helen Li, Ying Feng, Changhui Bao, Yanyuan Bai

**Affiliations:** ^1^School of Foreign Languages, Wuhan University of Technology, Wuhan, China; ^2^Wuluolu Middle School, Wuhan, China

**Keywords:** language attrition, lexical attrition, attained proficiency, length of exposure, dynamic systems theory

## Abstract

Compared to the considerable scholarly focus on language acquisition, research into language attrition has not received as much attention. Current studies have tended to emphasize either linguistic or extralinguistic factors impacting language attrition, rather than examining how these various factors might interact with one another. Consequently, it remains unclear whether there are any interactional effects among the different contributing elements of language attrition. This paper explored the relationships between attained proficiency and duration of exposure through a vocabulary assessment conducted with going-to-be sophomores (*n* = 110). The findings indicated that the attained proficiency served as an initial factor, while the lengths of exposure to a foreign language reflected the extent of language attrition. Additionally, different types of exposure were also found to be influential for lexical attrition. These results provide valuable insights for second language pedagogy and help enhance students’ capabilities in acquiring foreign languages.

## 1 Introduction

Research conducted over the last 40 years on first language (L1) attrition and second or foreign language (L2) attrition has primarily documented the nature of language attrition (what it is), the attrition of language skills (how it happens), and the influencing factors for such attrition (why it happens).

To start with, studies investigating into the nature of language attrition can be traced back to the 1980s during which language attrition was characterized as a phenomenon where individuals or speech communities experience a decline in language proficiency as one language or any portion of one language was gradually supplanted by another (Freed and Lambert, 1982). While early studies viewed language attrition as language shift, language regression, language loss, and language death (de Bot and Weltens, 1995; Freed and Lambert, 1982; Grenoble and Whaley, 1996; Hyltenstam and Viberg, 1993; Freed and Lambert, 1982; Seliger and Vago, 1991; Silva-Corvalán, 1991), more recent research interpreted it as a gradual reduction or loss of linguistic knowledge and skills in an individual when there was a lack of use in bilingual or multilingual contexts (de Bot, 2000; Köpke, 2001; Köpke and Nespoulous, 2001; Ni, 2007; Park, 2018; Schmid, 2008, 2009, 2013; Zhang, 2023). Unlike earlier definitions that emphasized a more social and pathological perspective, contemporary definitions focus on individual and non-pathological aspects. For instance, one such definition describes it as “the non-pathological decrease in proficiency in a language that had previously been acquired by an individual” (Köpke and Schmid, 2004, p. 3). In this vein, language attrition, by its very nature, encompasses a variety of intricate relationships, reflecting the changes in the dynamic interplay among an individual’s languages across all linguistic domains and through various modalities of production, processing, and understanding (Schmitt and Sorokina, 2024).

In addition, studies addressing attrition of language skills concur that varying levels of attrition can occur across different language domains, such as speaking (e.g., de Leeuw et al., 2023; Mayr et al., 2020), listening (e.g., Karlin and Karlin, 2018; Yu and Chan, 2009), reading (e.g., Alharthi and Al Fraidan, 2016; Deng, 2023), and writing (e.g., Asgari, 2013; Heng et al., 2012; Mytara and Köpke, 2024). More importantly, vocabulary—a subskill of these language skills, is particularly influenced by the duration of time. It is easily altered, and vulnerable to the impacts of foreign language attrition in contrast to phonological, morphological, and syntactic knowledge (Hu and Kouider, 2024; Ni and Jin, 2020; Zhang, 2023). Therefore, lexical attrition, an important factor often highlighted by attrition researchers (e.g., de Bot and Weltens, 1995; Gallo et al., 2021; Goral et al., 2008; Hu, 2023; Schmid, 2006, 2011), is viewed as the phenomenon of language attrition that is the most susceptible to occurrence (Zhang, 2023). It serves as the primary focus in the study of language attrition, which has laid the groundwork for examining other elements such as phonetics, semantics, and syntax, and thus merits further investigation (Hu and Kouider, 2024).

At last, studies exploring why language attrition happens have identified multiple contributing factors from both linguistic and extralinguistic aspects, including frequency and patterns of language use (e.g., Park, 2018), the use of and exposure to L1 and L2 (e.g., Silva and Arantes et al., 2021; Yang, 2023), language proficiency (e.g., Asgari and Mustapha, 2012; Zhang, 2023), memory and attention (e.g., Bylund et al., 2010; Schmid et al., 2022), age (e.g., Ahn et al., 2017), attitudes toward L1 and L2 (e.g., Cherciov, 2012; Kasap, 2020), aptitude (e.g., Mackey, 2022), gender (e.g., Anderson, 1999; Li, 2016; Ni, 2009b), identity (e.g., Schmid, 2004, 2011), motivation (e.g., Grosjean et al., 2013), integration and affiliation (e.g., Schmid and Cherciov, 2019), cultural influences (e.g., Riaz et al., 2021; Schmid and Dusseldorp, 2010; Titone and Tiv, 2023), and neuroimaging elements (Gallo et al., 2021). It is crucial to understand that language attrition results from the interaction of multiple influences; no single factor can fully account for this phenomenon (Hu and Kouider, 2024). While many of these studies have examined various factors influencing language attrition, few of them have probed into the effects of the interplay of different factors on language attrition.

In summary, recent studies have highlighted that language attrition is a complex and dynamic process of language changes, which is non-pathological and focuses on the individual. Furthermore, among the attrition of various language skills, vocabulary has generally been considered as one of the most vulnerable domains impacted by language attrition. Finally, current studies have favored linguistic or extralinguistic factors impacting language attrition, rather than examining how these various factors might interact with one another. The present study thus aims to unveil whether there are any interactional effects among the different contributing elements of language attrition, particularly in the most susceptible domain—lexical attrition.

## 2 Literature review

### 2.1 Lexical attrition

Vocabulary constitutes the words and their meanings in a given language (Hu and Kouider, 2024). It is an essential element of linguistic knowledge and serves as a crucial instrument for effective communication. Attrition of vocabulary, or lexical attrition, refers to the decline in vocabulary proficiency that occurs when language learners stop their efforts to learn the language (Ni, 2009a). It encompasses the decline in lexical meaning, the diminished capacity for semantic differentiation, and a reduction in vocabulary usage and expressive ability. Given the crucial importance of vocabulary in language acquisition, the mastery of vocabulary is considered as a strong predictor of overall proficiency in that language (Montrul, 2009). As a result, researchers have shown considerable interest in investigating lexical attrition, with a specific focus on the factors that influence L2 lexical attrition (e.g., Alharthi, 2014a; Alharthi, 2014b; Ghasemi Bagherabadi, 2005; Hu and Kouider, 2024; Jessner et al., 2021; Morshedian, 2008; Ni and Jin, 2020; Schmitt and Sorokina, 2024; Titone and Tiv, 2023).

Early studies in L2 contexts have concentrated on linguistic factors contributing to lexical attrition, such as part of speech, word length, and frequency, etc. For example, Ghasemi Bagherabadi (2005) examined the loss of English vocabulary during summer vacation among 25 Iranian high school students. The findings indicated that verbs and adjectives were more prone to attrition compared to nouns, and that vocabulary acquired at the start of the semester was retained more effectively than that learned toward the end of the semester. Morshedian (2008) carried out an empirical investigation into the loss and maintenance of both productive and receptive vocabulary among Iranian learners of foreign languages. The results revealed that, after a three-month period without English usage, there was a notable decline in the participants’ productive vocabulary in contrast to their receptive vocabulary. Similarly, Alharthi (2014a) noted that EFL students in Saudi Arabia experienced a decrease in vocabulary retention upon finishing their studies. This decline was attributed to their limited exposure to the English language outside of university courses, where they primarily encountered it. The research highlighted different aspects of language attrition by analyzing various parts of speech.

Recent studies in L2 have focused more on extralinguistic factors such as attrition time, gender, motivation, attitude, learning strategies, attained proficiency, length of exposure, and so on. Regarding the timing of attrition, several empirical researchers have identified a trend indicating that attrition takes place swiftly during the early stage, decreases in pace during the middle phase, and then speeds up once more in the later stages (e.g., Hansen, 1999; Ni and Yan, 2006; Wang and Li, 2015).

As for gender differences in language attrition, while scholars recognize its significance as an influencing factor, there is no consensus on whether males or females retain language skills more effectively. Ni (2009b) study showed that males experienced less attrition than females. Conversely, Lin (2015) reported that males exhibited a higher rate of attrition in listening and reading abilities compared to females but showed little difference in writing skills.

Motivation and attitude also significantly influence language attrition. Zhong and Sun (2012) identified personal learning motivation and emotional factors as key influences on lexical attrition. Similarly, other researchers (e.g., Kasap, 2021; Pan et al., 2023, 2024; Wang and Li, 2015) concurred that varying levels of learner motivation led to different rates of language attrition.

Learning strategies play a crucial role in influencing lexical attrition as well. Research by Kesmez (2021) demonstrated that the use of cognitive and metacognitive strategies—along with memory aids and social consolidation techniques—correlated with lexical retention. Alharthi (2014b) found that rote learning methods and note-taking strategies uniquely influenced learners’ retention of receptive versus productive vocabulary.

Meanwhile, attained proficiency serves as a dependable indicator of both retention and attrition across various attrition studies (e.g., Liu, 2010; Tracy-Ventura et al., 2025; Schmid, 2023). Tomiyama (2009) proved that attrition in lexical productivity was different between two siblings based on their storytelling data collected over the duration of 31 months. Mehotcheva (2010) explored college students’ attrition on Spanish as a foreign language, and Dutch or German as the native language. The study reported that among variables like exposure to language, attrition time, motivation (attitude), and proficiency, attained proficiency was the strongest predictor to lexical attrition against their native Dutch or German backgrounds. Xu (2010) also found that attained proficiency was a reliable indicator to lexical attrition for Chinese-Dutch language learners. Additionally, Asgari and Mustapha (2012) highlighted significant variations in abstract versus concrete noun attrition rates across different proficiency levels among non-continuing learners. Jessner et al. (2021) indicated that language usage was a less influential variable compared to initial proficiency level concerning language attrition. Collectively, these findings support the conclusion that attained proficiency is a strong predictor to lexical attrition, and the higher the level is, the better the language learners will perform after the formal instruction of learning.

Similarly, length of exposure has been posited by scholars as another strong potential variable affecting L2 attrition outcomes. Hansen (1999) along with Faraj and Hamid (2023), suggested that more frequent exposure to language led to a better attainment in that language. This implies that participants who regularly immerse themselves in a language and actively interact with it, are likely to stimulate the neural pathways associated with language comprehension and production. Such consistent engagement enhances their overall linguistic competence by reinforcing vocabulary retention through repeated activation in memory systems.

The results of the reviewed literature imply that while the languages that undergo attrition differ from one another, there are commonalities among the variables affecting them. Compared with the majority of attrition studies documenting various influencing factors, limited studies have addressed the interplay of different contributing variables to L2 lexical attrition (Fu, 2019; Gardner et al., 1985; Hu, 2023; Hu and Kouider, 2024; Jia and Aaronson, 2003; Kramer et al., 2021). In other words, current research in L2 often focuses on the impact of individual factors on lexical attrition rather than exploring the interaction between multiple influencing factors. Particularly, there has been a lack of studies in L2 examining the interplay between attained proficiency and exposure length—the two strong predictors to lexical attrition.

### 2.2 Dynamic systems theory

Dynamic Systems Theory (DST) has been recognized as an effective framework for understanding language attrition (de Bot, 2008; de Leeuw et al., 2013; Schmid et al., 2013). Central to DST are its emphasis on ongoing change and the interconnectedness of internal components (de Bot et al., 2007, de Bot and Larsen-Freeman, 2011; Freeman and Cameron, 2008; Schmid and Mehotcheva, 2012; van Geert, 1991). This focus renders it particularly suitable for theorizing and interpreting investigations on language attrition, given the dynamic, complex, non-pathological, and non-linear characteristics associated with this phenomenon (Li and Liu, 2024).

DST emphasizes that a system is perpetually evolving in a non-linear trajectory, which indicates that language development occurs through a nonlinear rather than a linear progression over time (Herdina and Jessner, 2013). This non-linear aspect is also apparent in language attrition, which is affected by multiple elements such as age, gender, prior language proficiency, exposure time, duration of non-use, as well as motivation and attitude toward learning and acquisition methods. However, not all these factors exert equal influence on language attrition. In situations like language attrition, these changes can be viewed as fluctuations within a language system that are continuously evolving and interrelated. Such a characteristic of DST aligns well with the non-linear, dynamic, and complex nature of language attrition, thus offering effective approaches to investigating and comprehending language attrition.

DST is also known as sensitive dependence on the initial conditions (Köpke, 2001). This concept highlights the unpredictability inherent in the process of language acquisition. As posited by DST, each system encompasses multiple subsystems that interact with one another (Bot et al., 2013; van Geert, 2008), ultimately leading to significant transformations within the entire system. In this sense, even minor variations in a system’s starting conditions can lead to significant differences within the entire system over time. To put it another way, various elements of language are interrelated and can influence one another rather than functioning independently, owing to the complexity of the language system involved. Recent research underpinned by DST has increasingly supported the presence of such interdependence or influence in language attrition. For example, difficulties with phonemic coding can impact not only reading and writing abilities but may also affect oral language development, which leads to the attrition of speaking skills, particularly in skills regarding the parts of listening and pronunciation (Sparks et al., 1995). Similarly, oral language skills may contribute to the decline of English reading proficiency because phonological awareness was proved to serve as a strong predictor for reading success across different languages (Giguere et al., 2024). In short, a slight modification in one aspect can have a considerable impact on other aspects within the language system. This feature provides a solid foundation to understand and interpret language attrition by examining the interplay of various contributing factors.

The review of the above pertinent literature regarding lexical attrition indicates that earlier research in L2 focused primarily on the linguistic factors affecting lexical attrition. Current researchers in L2, on the other hand, show a tendency to concentrate on extralinguistic factors. More significantly, both early and current research on L2 attrition has demonstrated a limited focus on the interplay of various factors. In particular, the relationship between attained proficiency and duration of exposure, the two significant factors influencing lexical attrition, has not received adequate attention. Finally, DST offers valuable insights into the process of language attrition, since its ongoing-evolving, non-linear, and interdependence nature well suits the dynamic, non-linear, and complex features of language attrition. Therefore, the current study intends to examine the interactions between attained proficiency and duration of exposure in the L2 context, and explore whether there exist any interactional effects between these two factors, particularly in the most susceptible domain—lexical attrition.

## 3 Methodology

### 3.1 Research questions

To tackle the identified research gaps, this study proposes three key questions as follows:

1. Does lexical attrition occur after the formal learning of English as a second language?
2. Do attained proficiency and duration of exposure contribute to lexical attrition of English as a second language?
3. Does there exist any relationships between the attained proficiency and the duration of exposure to lexical attrition of English as a second language?

### 3.2 Participants

The participants were obtained using a convenience sampling approach. The dataset consisted of 110 students preparing to enter their sophomore year from a university in central China. Due to the convenience of accessing foreign language students, most of the participants (*N* = 75) were sampled from the foreign language majors. Additionally, to enhance the validity and reliability of the study, an extra group of participants (*N* = 35) was included. Statistical data indicates that the participants hail from 26 provinces across China, with most originating from Hubei province, followed by Hunan and Zhejiang provinces.

From the examination of these 110 datasets, it was evident that regarding age distribution, a significant portion of the sample (54.5%) was comprised of individuals aged 19, aligning with their status as future sophomores. Concerning gender distribution, females constituted a larger share of the sample at 66.4%, compared to males at 33.6%. As for academic major distribution, there was a relatively balanced representation across various fields of study, with English majors constituting the largest share at 37.3%, followed by Materials at 31.8% (Table 1).

**Table 1:** Sample distribution analysis.

Question items	Option	Number of participants	Account for (%)
Age	18	30	27.3
	19	60	54.5
	20	18	16.4
	21	1	0.9
	24	1	0.9
Gender	Man	37	33.6
	Woman	73	66.4
Major	Materials	35	31.8
	French	23	20.9
	Japanese	11	10.0
	English	41	37.3

### 3.3 Instrument

In this study, two assessments were given to the 110 going-to-be sophomore students. The initial assessment served as a pre-test. It not only included essential information such as the participants’ names, ages, genders, and majors, but also comprised 100 multiple-choice questions centered on vocabulary from CET-4 (College English Test Band 4) that students had previously learned. The vocabulary items from CET-4 were selected randomly, without considering factors such as word frequency, speech of word, origin of word, or length of word. This method allows students to undertake a relatively fair and balanced assessment under these unconstrained conditions. The follow-up assessment served as a post-test. It retained the same set of 100 questions but introduced additional inquiries regarding participants’ engagement with English during the summer holiday. These included: the frequency of their exposure to English over the vacation; the methods by which they engaged with the language; and their motivations for maintaining contact with English.

The pre-test was carried out in July 2024, shortly after students completed their formal education for the first year. The post-test took place in September 2024, approximately 2 months later, which was at the beginning of the students’ sophomore year. Both assessments were designed using *Wen Juan Xing*—a professional and widely-used online survey platform in China. Participants accessed these tests through QR code scanning.

All student participants consented to complete the tests independently without utilizing any external resources. To ensure the accuracy of the assessments, each student was presented with five options to choose from; four of these were Chinese translations of the vocabulary, while the fifth option was “Unsure.” Furthermore, to alleviate students’ anxiety, they were informed that the tests were solely for research purposes and did not evaluate their individual abilities.

### 3.4 Data analysis

The analysis of the data was performed using SPSS Statistics 27. Firstly, normality was examined prior to statistical analyses. The dataset exhibited a normal distribution, as evidenced by the standardized Skewness and Kurtosis falling within the range of |3| and |8| (Field, 2009). Additionally, Cronbach’s Alpha (Cronbach, 1951) was recorded at 0.912, reflecting high consistency or reliability within the scale items. Furthermore, the Kaiser–Meyer–Olkin (KMO) (Kaiser, 1974) turned out to be 0.219, suggesting that the variables might not be conducive for factor analysis. Consequently, alternative analytical methods were employed, including paired sample tests, paired *t*-tests, and one-way analysis of variance. At last, the Bartlett’s test (Bartlett, 1937) for sphericity yielded an approximate chi-square value of 9356.558 with 4,950 degrees of freedom and a significance level less than 0.001. Since this significance level was below 0.05, the null hypothesis was rejected, thereby confirming a significant correlation between the examined variables.

During the analyses, a paired test was utilized to determine if lexical attrition occurred after the summer holiday. Pearson Correlation analyses and paired tests were then employed to examine the relationship between attained proficiency levels and the overall degree of lexical attrition. Further Pearson Correlations were conducted to investigate how exposure to English correlated with the degree of lexical attrition. Moreover, paired sample *t*-tests were performed to analyze lexical attrition at specific proficiency levels and amounts of exposure, respectively. Ultimately, a single-factor analysis of variance was performed to compare differences in attrition among participants exposed to different forms of English interaction.

After approximately 2 months of the summer holiday, 58 out of 110 students showed signs of language attrition, leading to an attrition rate of 52.7%. Consequently, the following results primarily concentrated on analyzing data from these 58 participants.

## 4 Results and discussion

### 4.1 Lexical attrition concerning different proficiency levels

In this study, students were classified into three distinct proficiency groups based on their pre-test results. Those scoring above 90, totaling 10 participants, were categorized as high-level students. The medium-level group consisted of 28 participants who achieved scores ranging from 70 to 90. Lastly, the low-level category included 20 participants whose scores fell below 70.

As illustrated in Table 2, the mean difference showed that the average score of the post-test (65.38) was significantly lower than that of the pre-test (74.29), with the significance level (2-tailed) below 0.001. Such a result reflected the existence of lexical attrition among the going-to-be sophomore student participants.

**Table 2:** Paired samples test of the pre-test and post-test.

Test	Mean	N	Min	Max	SD	Std. error mean	*t*	Sig. (2-tailed)
Pre-test	74.29	58	20	100	18.615	2.444	8.609	<0.001
Post-test	65.38	58	8	99	25.255	3.316		

Furthermore, the average English proficiency score at the outset for these individuals was 74.29, with a standard deviation of 18.615, suggesting variability in their English proficiency levels (see Table 3). The mean lexical attrition value was 8.74, accompanied by a standard deviation of 7.400, reflecting notable individual differences in lexical attrition. The correlation coefficient (R) between initial proficiency and the overall degree of lexical attrition was −0.801, signifying a negative relationship. In particular, individuals with higher initial English proficiency tended to experience lower levels of lexical attrition, while those with lower initial proficiency incurred greater lexical attrition. The two-tailed significance level was below 0.001, indicating an inverse correlation between initial proficiency and lexical attrition degree (see Table 3).

**Table 3:** Correlation between initial proficiency and lexical attrition degree.

Factors	N	Mean	SD	*R*	Sig.(2-tailed)
Initial proficiency	58	74.29	18.615	−0.801	<0.001
Overall lexical attrition degree	58	8.74	7.400		

The data on lexical attrition among students categorized by high, intermediate, and low English proficiency levels is detailed in Table 4. Students classified as having high proficiency achieved an average score of 94.60 in the pre-test, which decreased slightly to 91.70 in the post-test, reflecting a minor degree of lexical attrition. Their overall performances concerning both tests remained relatively high when compared to those of the other two student groups, signifying the lowest degree of lexical attrition (see Table 4). In contrast, the intermediate-level students got an average score of 81.93 in the pre-test, which dropped to 76.25 in the post-test, indicating a significant degree of lexical attrition. Meanwhile, those categorized as low-level learners started with an average score of 53.45 in the pre-test but showed a substantial fall to 37.00 in the post-test, representing the highest degree of lexical attrition among the three groups.

**Table 4:** Lexical attrition of students with different proficiency levels.

Level	Test	Mean	N	SD	Min	Max	*t*	Sig.
High proficiency	Pre-test	94.60	10	3.340	90	100	4.529	<0.001
	Post-test	91.70	10	5.272	84	99		
Intermediate proficiency	Pre-test	81.93	28	1.355	70	89	7.024	<0.001
	Post-test	76.25	28	1.865	55	89		
Low proficiency	Pre-test	53.45	20	14.177	20	69	9.170	<0.001
	Post-test	37.00	20	19.374	8	62		

This observed inverse relationship between achieved English proficiency and lexical attrition suggests that as proficiency increases, the likelihood of language attrition decreases. Such findings aligned with Mehotcheva’s (2010) study which reported that initial proficiency was “the most salient predictor of language retention with high proficiency at onset leading to better retention of the language” (p. 154). Similarly, Jessner et al. (2021) asserted that a higher level of initial proficiency was linked to enhanced retention of a foreign language, especially over the long term. Their findings suggested that individuals with lower initial proficiency tended to experience greater language attrition, which further emphasized the negative correlation between attained English proficiency and lexical attrition.

According to DST, language systems consist of various interrelated subsystems, which can result in considerable changes across the entire system. Consequently, even slight alterations in a language system’s initial conditions can produce substantial differences throughout the system as time progresses. In other words, individuals who possess higher language skills prior to experiencing attrition tend to demonstrate a lesser degree of language attrition.

### 4.2 Lexical attrition concerning different lengths of exposure

In order to explore whether different lengths of exposure have impact on lexical attrition, participants were divided into four groups according to their varying lengths of exposure to English during the summer holiday. These groups comprised students with frequent contact with English, those with occasional contact, those with limited contact, and a final group with no contact. The classification of these varying exposure lengths was based on participants’ self-reported assessments in the post-test, which included questions like “What was your frequency of English exposure during the summer vacation?,” and “What was your average exposure time per day in English?.” Specifically, participants with an average exposure time exceeding 60 min per day were categorized into the frequent contact group; those with exposure between 30 and 60 min were assigned to the occasional contact group; students who engaged for less than 30 min fell into the limited contact group; and individuals with minimal or no exposure were grouped as no-contact.

Concerning the group of students who had frequent contact with English, the correlation coefficient (R) was −0.594 and the significance value (Sig.) was below 0.001 (see Table 5). These results indicated that students who were frequently exposed to English experienced a comparatively lower rate of lexical attrition. For students who had occasional contact with English, the correlation coefficient (R) was −0.262 and the significance value was 0.047 (see Table 6). Such results implied that students who were occasionally exposed to English experienced a comparatively higher rate of lexical attrition compared with those who were frequently exposed to English. In regard to the group of students who had limited contact with English, the correlation coefficient (R) was −0.227 and the significance value was 0.087 (see Table 7). These results reflected that students who were exposed to English within limited time exhibited a significantly higher rate of lexical attrition compared with those who were frequently exposed to English and those who were occasionally exposed to English. The correlation coefficient for students who had no contact with English was −0.805 and the significance value was lower than 0.001 (see Table 8). Such results revealed that students who had no exposure to English during the summer holiday experienced the highest degree of lexical attrition.

**Table 5:** Correlation between frequent contact and overall lexical attrition degree.

Factors	N	Mean	SD	*R*	Sig.(2-tailed)
Frequent contact	16	0.28	0.451	−0.594	<0.001
Overall lexical attrition degree	16	8.74	7.400		

**Table 6:** Correlation between occasional contact and overall lexical attrition degree.

Factors	N	Mean	SD	*R*	Sig.(2-tailed)
Occasional contact	16	0.28	0.451	−0.262	0.047
Overall lexical attrition degree	16	8.74	7.400		

**Table 7:** Correlation between limited contact and overall lexical attrition degree.

Factors	N	Mean	SD	*R*	Sig.(2-tailed)
Limited Contact	18	0.31	0.467	−0.227	0.087
Overall lexical attrition degree	18	8.74	7.400		

**Table 8:** Correlation between no-contact and overall lexical attrition degree.

Factors	N	Mean	SD	*R*	Sig.(2-tailed)
No-contact	8	0.14	0.348	−0.805	<0.001
Overall lexical attrition degree	8	8.74	7.400		

The identified negative correlation between lengths of exposure and lexical attrition corresponded with the results from previous research conducted by Włosowicz (2015), Chaouch-Orozco and Martin-Villena (2024), as well as Matos and Flores (2024). These studies collectively acknowledged the significant influence of language contact or exposure on language attrition.

In summary, frequent engagement with English appears to mitigate lexical attrition; hence, increased frequency leads to reduced attrition rates. The findings indicate that students who frequently encounter English have a diminished activation threshold for their lexical abilities in that language, which helps enhance their vocabulary skills and leads to less language attrition. To view it through the lens of DST, various forms of linguistic knowledge require varying levels of stimulus for activation. Thus, for learners wishing to preserve more vocabulary, it is beneficial to increase their exposure to that language. In other words, language instructors are suggested to immerse students in a foreign language environment, expose them to the culture associated with the language, and reinforce the use of vocabulary as frequently as possible.

### 4.3 Correlation between attained proficiency and length of exposure

Table 9 presented the influence of different lengths of exposure to lexical attrition under the same degree of attained proficiency. In terms of statistical significance, within the frequent contact group at the high proficiency level, a *t*-value of −2.967 and a *p*-value of 0.016 revealed a notably reduced rate of lexical attrition. The occasional contact group showed a significant difference with a t-value of −4.628 and a *p*-value below 0.001, reinforcing the pattern of diminished attrition rates tied to increased exposure. Similarly, within the limited contact group, the *t*-value was −4.583 and the *p*-value was below 0.001, further emphasizing the negative correlation between exposure and lexical attrition for individuals at the same proficiency level. As for the no-contact group, the *t*-value was −4.529 and the *p*-value was below 0.001, indicating that there existed a connection between exposure duration and lexical attrition when prior proficiency was taken into consideration.

**Table 9:** The influence of different lengths of exposure to lexical attrition under the same degree of attained proficiency.

Proficiency	Group	Mean	N	SD	Min	Max	*t*	Sig.
High proficiency	Frequent contact	0.60	6	0.516	0	1	−2.967	0.016
	Lexical attrition degree	2.90	6	2.025	0	6		
	Occasional contact	0.30	3	0.483	0	1	−4.628	<0.001
	Lexical attrition degree	2.90	3	2.025	0	6		
	Limited contact	0.10	1	0.316	0	1	−4.583	<0.001
	Lexical attrition degree	2.90	1	2.025	0	6		
	No-contact	0.00	0	0.00	0	0	−4.529	<0.001
	Lexical attrition degree	2.90	0	2.025	0	6		
Intermediate proficiency	Frequent contact	0.36	10	0.488	0	1	−6.075	<0.001
	Lexical attrition degree	5.68	10	4.278	0	15		
	Occasional contact	0.39	11	0.497	0	1	−6.488	<0.001
	Lexical attrition degree	5.68	11	4.278	0	15		
	Limited contact	0.25	7	0.441	0	1	−7.293	<0.001
	Lexical attrition degree	5.68	7	4.278	0	15		
	No-contact	0.00	0	0.00	0	0	−7.024	<0.001
	Lexical attrition degree	5.68	0	4.278	0	15		
Low proficiency	Frequent contact	0.00	0	0.00	0	0	−10.10	<0.001
	Lexical attrition degree	15.95	0	7.060	7	29		
	Occasional contact	0.10	2	0.308	0	1	−9.847	<0.001
	Lexical attrition degree	15.95	2	7.060	7	29		
	Limited contact	0.50	10	0.513	0	1	−9.353	<0.001
	Lexical attrition degree	15.95	10	7.060	7	29		
	No-contact	0.40	8	0.503	0	1	−10.52	<0.001
	Lexical attrition degree	15.95	8	7.060	7	29		

At the intermediate proficiency level, both frequent and occasional contact groups reflected significant declines in lexical attrition rates corresponding to increased exposure. Meanwhile, anticipated trends for groups with limited or no contact revealed rising attrition rates alongside decreasing exposure levels. In other words, for learners at the intermediate level, greater exposure to English correlated positively with lexical attrition, denoting that increased contact led to reduced attrition rates.

As for the low proficiency level, the *t*-values for frequent contact (−10.10), occasional contact (−9.847), limited contact (−9.353), and no-contact (−10.52) supported the notion that at certain proficiency levels, increased exposure to a foreign language correlated with reduced lexical attrition. Meanwhile, all *p*-values for the low proficiency group were below 0.001, thereby reinforcing the relationship between proficiency levels and exposure to vocabulary. More importantly, when compared to high and intermediate proficiency levels, the *t*-values for the low proficiency group were greater than those of the other two groups. This indicated that individuals with a lower command of vocabulary, regardless of their duration of exposure to the language, were significantly more susceptible to experiencing a higher degree of lexical attrition.

Table 10 showed how various proficiency levels impacted lexical attrition when exposure remained constant. In cases of frequent contact with English, participants with high proficiency demonstrated minimal lexical attrition (*M* = 1.67), while those at the intermediate level showed slightly higher attrition (*M* = 1.70). The standard deviation of lexical attrition for the high proficiency group was 1.366, compared to 1.567 for the intermediate group, highlighting differing rates of lexical attrition among the participants. Conversely, due to an insufficient sample size (*N* = 0), there was no variability in the standard deviation at the low proficiency level, indicating that individuals frequently exposed to English at this level displayed consistent lexical attrition rates.

**Table 10:** The influence of different proficiency levels to lexical attrition under the same degree of exposure.

Group	Level	Mean	N	SD	Min	Max	*t*	Sig.
Frequent contact	High proficiency	96.50	6	2.588	93	100	60.863	<0.001
	Lexical attrition degree	1.67	6	1.366	0	4		
	Intermediate proficiency	86.50	10	2.953	80	89	75.479	<0.001
	Lexical attrition degree	1.70	10	1.567	0	4		
	Low proficiency	0	0	0	0	0		
	Lexical attrition degree	0	0	0	0	0		
Occasional contact	High proficiency	92.33	3	2.082	90	94	43.237	<0.001
	Lexical attrition degree	4.67	3	1.528	3	6		
	Intermediate proficiency	81.18	11	7.808	70	89	30.458	<0.001
	Lexical attrition degree	5.64	11	1.502	3	8		
	Low proficiency	68.00	2	1.414	67	69	61.000	0.010
	Lexical attrition degree	7.00	2	0.000	7	7		
Limited contact	High proficiency	90.00	1	0	0	0		
	Lexical attrition degree	5.00	1	0	0	0		
	Intermediate proficiency	76.57	7	6.876	70	89	27.316	<0.001
	Lexical attrition degree	11.43	7	3.155	8	15		
	Low proficiency	59.00	10	13.936	20	66	10.720	<0.001
	Lexical attrition degree	11.70	10	1.337	10	14		
No-contact	High proficiency	0	0	0	0	0		
	Lexical attrition degree	0	0	0	0	0		
	Intermediate proficiency	0	0	0	0	0		
	Lexical attrition degree	0	0	0	0	0		
	Low proficiency	42.88	8	7.954	27	52	7.865	<0.001
	Lexical attrition degree	23.50	8	4.375	17	29		

When evaluating conditions of occasional contact with English, it was observed that the group with high proficiency experienced lower levels of lexical attrition (*M* = 4.67). In contrast, the intermediate proficiency group demonstrated a marginally higher level of lexical attrition (*M* = 5.64). The low proficiency group displayed the highest rate of lexical attrition (*M* = 7.00), underscoring significant disparities in the rates of lexical attrition among the participants.

Under circumstances of limited contact with English, the presence of only one sample within the high proficiency level group precluded the possibility of performing paired sample analyses. Nonetheless, it was clear that the intermediate proficiency group exhibited a relatively low rate of lexical attrition (*M* = 11.43), whereas the low proficiency group demonstrated a higher rate of lexical attrition (*M* = 11.70).

In conditions with no exposure to English, the high and intermediate proficiency groups had no samples, while the low proficiency group experienced a significantly greater level of lexical attrition (*M* = 23.50), highlighting the statistical significance of this discrepancy.

Despite the extremely limited sample sizes (i.e., no participants or just one participant in Tables 9, 10), this study still deemed such data relevant for several reasons. Firstly, including these data helped maintain the integrity of the dataset and enhanced the overall scope of the research. Secondly, analyzing all available data mitigated potential selection bias (Winship and Mare, 1992), which is crucial for ensuring that results remain objective. Lastly, there was a consideration to prevent sample waste (Ioannidis et al., 2014) while optimizing the value derived from the data. Consequently, even cases with *N* = 0 or *N* = 1 may provide essential insights; omitting them could lead to imprecise results (Reio, 2016) when assessing lexical attrition.

In summary, Tables 9, 10 demonstrated a relationship between achieved proficiency and different lengths of exposure to English. Such an interplay is consistent with the Dynamic Systems Theory, which views language as a multifaceted system made up of several interconnected subsystems, where small alterations can impact overall results. The nature of this interconnection is further explored by analyzing how attained proficiency interacts with the length of exposure.

DST posits that language acquisition is an ongoing evolving process, where various factors in language learning are interconnected. This inherent dynamism of the language system can be analyzed from two perspectives. Firstly, every individual learner possesses a unique language system, and these diverse systems interact with one another, resulting in dynamic variability influenced by external factors. Secondly, the different elements within each learner’s internal language system also interact and undergo continuous changes, reflecting the impact of internal variables and demonstrating interconnected variability.

Underpinned by DST, this study revealed that participants who possessed a relatively high level of attained proficiency and frequent contact with English showed minimal attrition rates. These participants appeared to have reached a stage of fossilization compared to their peers, thus allowing them to retain vocabulary more effectively. Such findings confirmed that higher levels of proficiency could mitigate lexical attrition and more exposure to foreign language could also decline the rate of lexical attrition. In other words, initial language proficiency prior to attrition and length of exposure were vital predictors to factors affecting lexical attrition.

Therefore, to effectively engage with the interactions and influences present in individual learners’ language systems, educators need to implement a flexible teaching strategy. This involves customizing instruction to suit students’ diverse abilities and providing experiences that address their unique characteristics and needs. Regarding the dynamics within each learner’s internal language system, teachers should encourage students to explore learning methods that align with their personal preferences.

### 4.4 Lexical attrition concerning different types of exposure

Aside from proficiency levels and lengths of exposure, different types of exposure to English as a second language also had impacts on the degree of lexical attrition.

According to Table 11, even though the *p*-value exceeded 0.005, it was still worthwhile to investigate the possible impacts of various exposure types on lexical attrition by examining the means and standard deviations. The mean score for listening was 11.18 and the standard deviation was 8.340, resulting in an *F*-value of 1.071. This relatively elevated mean for listening suggested that participants who primarily engaged with English through listening faced a notable rate of lexical attrition. In comparison, the mean score for speaking was 6.50 and the standard deviation was 6.552, indicating that participants who practiced speaking English during their summer vacation experienced a lower level of lexical attrition. The mean score for reading was 10.53 and the standard deviation was 8.323. Such a relatively higher level of lexical attrition was similar to the one observed in listening, implying significant lexical attrition in reading as well. Writing achieved a mean score of 7.50 with a standard deviation of 8.888, placing it at a moderate level and suggesting that writing-related attrition may be situated between the levels found in speaking and those seen in reading or listening contexts. Finally, translation had a mean value of 7.10 and a standard deviation of 4.748. The low scores aligned closely with those from speaking, suggesting that experiences related to translation might result in minimal lexical attrition.

**Table 11:** Correlation between different types of exposure and overall lexical attrition degree.

Types of Exposure		N	Mean	SD	*F*	Sig(2-tailes)
Listening	49	11.18	8.340	1.071	0.380
Speaking	26	6.50	6.552		
Reading	40	10.53	8.323		
Writing	27	7.50	8.888		
Translation	18	7.10	4.748		

In summary, lexical attrition was observed to be more significant in listening and reading than in speaking and translation, where it appeared to be less intense. The results indicated that students who practiced speaking and participated in translation exercises showed a stronger ability to resist lexical attrition. Therefore, learners who took part in speaking tasks and translation activities were likely to maintain their English vocabulary more effectively than those who primarily focused on listening or reading.

## 5 Conclusion and implications

This study examined the correlation between attained proficiency and the length of exposure, along with their effects on lexical attrition among Chinese college EFL students. To achieve this, a vocabulary assessment with a pre-test and a post-test was administered to the going-to-be sophomore students. The pre-test was administered at the beginning of students’ summer vacation and the post-test was conducted at the end of this approximately two-month holiday.

By analyzing the results from both the pre-test and post-test, the study identified that lexical attrition occurred during the summer vacation, with a language attrition rate of 52.7% (calculated as 58÷110 × 100%). Additionally, it was established that prior English proficiency and the duration of exposure to the foreign language were significant factors affecting lexical attrition. Finally, different types of exposure also influenced the degree of lexical attrition. The relationship between attained proficiency and exposure duration was examined through two paired sample t-tests by treating these factors as independent variables individually. This approach demonstrated that the attrition process among students exhibited key characteristics associated with DST.

The study further provided empirical evidence that college EFL students experienced lexical attrition after a summer break of nearly 2 months. Consequently, it is advisable for teachers to motivate learners to retain a larger portion of the vocabulary acquired in school, taking into account the impact of their language proficiency and exposure to English. Although students with high proficiency and frequent contact with English still face some degree of lexical attrition, it is evident that their rate of lexical attrition is significantly lower compared to those at intermediate and low proficiency levels with limited exposure to the language. Therefore, English instructors should focus on improving their students’ foundational skills and increasing opportunities for language exposure. For instance, teachers are encouraged to organize students into various classes based on assessments conducted immediately upon their return for the new semester. Furthermore, language learners themselves can mitigate the effects of lexical attrition by striving to achieve a high level of English proficiency prior to experiencing any breaks in formal instruction. They should also adopt a “lifelong learning” mindset to continue their English studies even they have finished their formal education.

Although this study explored the interplay between attained proficiency and length of exposure, both factors were recognized as extralinguistic influences on language attrition. Future research could delve deeper into the relationships between linguistic and extralinguistic elements. In addition, the design of the vocabulary test was limited, employing only multiple-choice questions for students in both the pre-test and post-test phases, which overlooked other question formats and led to a relatively low KMO value. Therefore, it is advisable for subsequent studies to address more diverse aspects of vocabulary. Finally, categorizing students into four distinct groups based on their self-reported daily exposure to English was somewhat subjective. This subjectivity could pose challenges for future researchers attempting to replicate similar studies. Thus, it is recommended that future research can document participants’ exposure time to a foreign language in a more objective manner.

## Data Availability

The original contributions presented in the study are included in the article/Supplementary material, further inquiries can be directed to the corresponding author.
