# Supplementary material for: Interactions between attained proficiency and length of exposure to lexical attrition of English as a second language
Source: Front Psychol. 2025 Sep 17;16:1586722. doi: 10.3389/fpsyg.2025.1586722 (PMC12483873; doi:10.3389/fpsyg.2025.1586722)
Supplement: Supplementary file 1 [file Data_Sheet_1.PDF]

Appendices

Appendix 1: Vocabulary Test Among College Students- Based on CET-4

This test is designed to evaluate the vocabulary volume of going-to-be sophomores, with a total of 100 questions, which may last for 10 minutes. Thanks for your cooperation. Please notice that when you are encountered with words which you are not sure about, please choose option E (unsure) for the accuracy of the test.

In addition, the information you fill in is all in the strictest confidence, and will only be used for research and analysis. I am here to promise that I will protect your privacy and security, and will not reveal it to any third party.

Name： [Fill in the blank] *

____

Age： [Fill in the blank] *

____

Major ： [Fill in the blank] *

____

1. Temporary [Multiple choice] *

A. Transient (Correct answer)

B. Temperature

C. Contemporary

D. Awesome

E. Unsure

2. Initiative [Multiple choice] *

A. Instead

B. Proposal (Correct answer)

C. Individual

D. Inactive

E. Unsure

3. Utilize [Multiple choice] *

A. Insignificant

B. Benthamism

C. Avail (Correct answer)

D. Ill-looking

E. Unsure

4. Subsequent [Multiple choice] *

A. Consequent (Correct answer)

B. Devastating

C. Substituted

D. Conquered

E. Unsure

5. Amateur [Multiple choice] *

A. Frustrated

B. Nonprofessional (Correct answer)

C. Accumulation

D. Mellow

E. Unsure

6. Abandon [Multiple choice] *

A. Sufferance

B. Ability

C. Ample

D. Discard (Correct answer)

E. Unsure

7. Withstand [Multiple choice] *

A. Retreat

B. Resist (Correct answer)

C. Intelligence

D. Understand

E. Unsure

8. Inflation [Multiple choice] *

A. Influence

B. Expansion (Correct answer)

C. Notice

D. Information

E. Unsure

9. Justify [Multiple choice] *

A. Certify (Correct answer)

B. Right

C. Judge

D. Travel

E. Unsure

10. Grind [Multiple choice] *

A. Grief

B. Grasp

C. Fragmentize (Correct answer)

D. Giant

E. Unsure

11. Halt [Multiple choice] *

A. Passage

B. Half

C. Stop (Correct answer)

D. Loath/ hate

E. Unsure

12. Seminar [Multiple choice] *

A. Service

B. Severe

C. Seminary

D. Symposium (Correct answer)

E. Unsure

13. Synthetic [Multiple choice] *

A. Artificial (Correct answer)

B. System

C. Symbol

D. Static

E. Unsure

14. Collapse [Multiple choice] *

A. Collude

B. Fall (Correct answer)

C. Collate

D. Dismantle

E. Unsure

15. Episode [Multiple choice] *

A. Expect

B. Express

C. Exercise

D. Series (Correct answer)

E. Unsure

16. Moisture [Multiple choice] *

A. Wetness (Correct answer)

B. Muzzle

C. Mystery

D. Instance

E. Unsure

17. Humble [Multiple choice] *

A. Unassuming (Correct answer)

B. Humorous

C. Hankering

D. Charitable

E. Unsure

18. Nuisance [Multiple choice] *

A. Numerous

B. Nature

C. Annoyance (Correct answer)

D. Nurse

E. Unsure

19. Stripe [Multiple choice] *

A. Naval

B. Strip

C. Variety (Correct answer)

D. Strike

E. Unsure

20. Fatigue [Multiple choice] *

A. Tiredness (Correct answer)

B. Fate

C. Coddle

D. Fair

E. Unsure

21. Accelerate [Multiple choice] *

A. Hasten (Correct answer)

B. Accent

C. Accept

D. Proximity

E. Unsure

22. Frustrate [Multiple choice] *

A. Member

B. Discouraging (Correct answer)

C. Melt

D. Fruit

E. Unsure

23. Penetrate [Multiple choice] *

A. Penalty

B. Pierce (Correct answer)

C. Episode

D. Practice

E. Unsure

24. Rigid [Multiple choice] *

A. Ride

B. Ridiculous

C. Upsurge

D. Tough (Correct answer)

E. Unsure

25. Dump [Multiple choice] *

A. Dumpling

B. Dumb

C. During

D. Fling/ trash (Correct answer)

E. Unsure

26. Recruit [Multiple choice] *

A. Entertain

B. Recent

C. Register (Correct answer)

D. Criticize

E. Unsure

27. Realm [Multiple choice] *

A. Domain (Correct answer)

B. Fact

C. Arrive

D. Dependence

E. Unsure

28. Saint [Multiple choice] *

A. Voyage

B. Sand

C. Deity (Correct answer)

D. Unitary

E. Unsure

29. Sensitive [Multiple choice] *

A. Sense

B. Delicate (Correct answer)

C. Adjudicative

D. Service

E. Unsure

30. Optical [Multiple choice] *

A. Choice

B. Optimistic

C. Opinion

D. Ocular (Correct answer)

E. Unsure

31. Demonstrate [Multiple choice] *

A. Democratic

B. Demand

C. Describe

D. Present (Correct answer)

E. Unsure

32. Accumulate [Multiple choice] *

A. Amass (Correct answer)

B. Accurate

C. Account

D. Actual

E. Unsure

33. Vibrate [Multiple choice] *

A. Village

B. Swing (Correct answer)

C. Vary

D. Vacation

E. Unsure

34. Tremendous [Multiple choice] *

A. Tendency

B. Treasure

C. Colossal (Correct answer)

D. Trace

E. Unsure

35. Sophisticated [Multiple choice] *

A. Spacious

B. Discreet

C. Complicated (Correct answer)

D. Sound

E. Unsure

36. Quotation [Multiple choice] *

A. Citation (Correct answer)

B. Quite

C. Qualification

D. Quiet

E. Unsure

37. Tropical [Multiple choice] *

A. Nutrient

B. Equatorial (Correct answer)

C. Storehouse

D. Malfunctioned

E. Unsure

38. Legislation [Multiple choice] *

A. Legality

B. Conceive

C. Insulate

D. Governance (Correct answer)

E. Unsure

39. Stale [Multiple choice] *

A. Suspend

B. Sleepy

C. State

D. Stinky (Correct answer)

E. Unsure

40. Barrier [Multiple choice] *

A. Obstruction (Correct answer)

B. Barrister

C. Barrel

D. Carrier

E. Unsure

41. Conscious [Multiple choice] *

A. Conscientious

B. Conservative

C. Prudent

D. Sentient (Correct answer)

E. Unsure

42. Flash [Multiple choice] *

A. Flesh

B. Flare

C. Flatter

D. Reflect (Correct answer)

E. Unsure

43. Breed [Multiple choice] *

A. Propagate (Correct answer)

B. Greed

C. Reed

D. Bread

E. Unsure

44. Intellectual [Multiple choice] *

A. Highbrow (Correct answer)

B. Intelligence

C. Institutional

D. Integrality

E. Unsure

45. Margin [Multiple choice] *

A. Marine

B. Edge (Correct answer)

C. Martyr

D. Swale

E. Unsure

46. Fulfill [Multiple choice] *

A. Accomplish (Correct answer)

B. Ideal

C. Irrespective

D. Improve

E. Unsure

47. Generous [Multiple choice] *

A. Moderate

B. Potent

C. Bounteous (Correct answer)

D. Innocent

E. Unsure

48. Insult [Multiple choice] *

A. Intrude

B. Explain

C. Instrument

D. Offend (Correct answer)

E. Unsure

49. Guarantee [Multiple choice] *

A. Hoggish

B. Associate

C. Pledge (Correct answer)

D. Instruct

E. Unsure

50. Indifferent [Multiple choice] *

A. Infantile

B. Unconcerned (Correct answer)

C. Inquire

D. Insist

E. Unsure

51. Opponent [Multiple choice] *

A. Inject

B. Springal

C. Hostile (Correct answer)

D. Superior

E. Unsure

52. Gown [Multiple choice] *

A. Frequency

B. Viewpoint

C. Frock (Correct answer)

D. Occasion

E. Unsure

53. Literary [Multiple choice] *

A. Library

B. Outlandish

C. Formal (Correct answer)

D. Announce

E. Unsure

54. Frontier [Multiple choice] *

A. Boundary (Correct answer)

B. Octagon

C. Imbed

D. Prescript

E. Unsure

55. Refine [Multiple choice] *

A. Appendage

B. Improve (Correct answer)

C. Previous

D. Question

E. Unsure

56. Combat [Multiple choice] *

A. Flame

B. Frog

C. Campaign (Correct answer)

D. Divide

E. Unsure

57. Capacity [Multiple choice] *

A. Spin

B. Disappointment

C. Exploitability

D. Competence (Correct answer)

E. Unsure

58. Starve [Multiple choice] *

A. Glossary

B. Infringe

C. Perish (Correct answer)

D. Corpse

E. Unsure

59. Cartoon [Multiple choice] *

A. Picture (Correct answer)

B. Ankle

C. Jargon

D. Freight

E. Unsure

60. Persist [Multiple choice] *

A. Restraint

B. Insist (Correct answer)

C. Elevator

D. Annals

E. Unsure

61. Fertile [Multiple choice] *

A. Fecund (Correct answer)

B. Futile

C. Hybrid

D. Fluent

E. Unsure

62. Freshman [Multiple choice] *

A. Immigration

B. Speech

C. Beginner (Correct answer)

D. Requirement

E. Unsure

63. Voyage [Multiple choice] *

A. Journey (Correct answer)

B. Snigger

C. Column

D. Impede

E. Unsure

64. Enhance [Multiple choice] *

A. Kettle

B. Wait

C. Disorient

D. Intensify (Correct answer)

E. Unsure

65. Exceed [Multiple choice] *

A. Dismantle

B. Practice

C. Surpass (Correct answer)

D. Opposition

E. Unsure

66. Perceive [Multiple choice] *

A. Contrive

B. Responsible

C. Detect (Correct answer)

D. Dandelion

E. Unsure

67. Cave [Multiple choice] *

A. Distort

B. Pit (Correct answer)

C. Settle

D. Transmission

E. Unsure

68. Handicap [Multiple choice] *

A. Obstacle (Correct answer)

B. Abundant

C. Requisite

D. Hole

E. Unsure

69. Jealous [Multiple choice] *

A. Envious (Correct answer)

B. Specialized

C. Vain

D. Unwilling

E. Unsure

70. Outlook [Multiple choice] *

A. Trauma

B. Purpose

C. Prospect (Correct answer)

D. Camara

E. Unsure

71. Dove [Multiple choice] *

A. Beach

B. Pigeon (Correct answer)

C. Self-esteem

D. Hailstone

E. Unsure

72. Flaw [Multiple choice] *

A. Call

B. Fault (Correct answer)

C. Dissolve

D. Breeze

E. Unsure

73. Privilege [Multiple choice] *

A. Peculiar (Correct answer)

B. Hummer

C. Mitigate

D. Accompanist

E. Unsure

74. Browse [Multiple choice] *

A. Garage

B. Aroma

C. Scan (Correct answer)

D. Resolve

E. Unsure

75. Addiction [Multiple choice] *

A. Admittance

B. Indulgence (Correct answer)

C. Committee

D. Avoidance

E. Unsure

76. Gather [Multiple choice] *

A. Evacuate

B. Collect (Correct answer)

C. Birth

D. Generate

E. Unsure

77. Riot [Multiple choice] *

A. Era

B. Invade

C. Violence (Correct answer)

D. Costa

E. Unsure

78. Bush [Multiple choice] *

A. Consent

B. Shrub (Correct answer)

C. Deficit

D. Meditate

E. Unsure

79. Wealth [Multiple choice] *

A. Property (Correct answer)

B. Health

C. Duck

D. Fineness

E. Unsure

80. Instruct [Multiple choice] *

A. Notebook

B. Train (Correct answer)

C. Argue

D. Demonstrate

E. Unsure

81. Dignity [Multiple choice] *

A. Jazz

B. Quantification

C. Sweater

D. Sanctity (Correct answer)

E. Unsure

82. Scan [Multiple choice] *

A. Skim (Correct answer)

B. Washroom

C. Reckon

D. Legend

E. Unsure

83. Cube [Multiple choice] *

A. Conviction

B. Solid (Correct answer)

C. Enigma

D. Longevity

E. Unsure

84. Supervise [Multiple choice] *

A. Oversee (Correct answer)

B. Doubt

C. Liquefaction

D. Nursing

E. Unsure

85. Remedy [Multiple choice] *

A. Queen

B. Therapy (Correct answer)

C. Trunk

D. Ready

E. Unsure

86. Intake [Multiple choice] *

A. Rose

B. Recommend

C. Entangle

D. Inhalation (Correct answer)

E. Unsure

87. Enthusiasm [Multiple choice] *

A. Increasement

B. Contract

C. Passion (Correct answer)

D. Implore

E. Unsure

88. Grasp [Multiple choice] *

A. Commute

B. Hinder

C. Catch (Correct answer)

D. Plantation

E. Unsure

89. Skeleton [Multiple choice] *

A. Bone (Correct answer)

B. Mercy

C. Broadcast

D. Habitant

E. Unsure

90. Cling [Multiple choice] *

A. Adhere (Correct answer)

B. Blame

C. Lurch

D. Example

E. Unsure

91. Cliff [Multiple choice] *

A. Bless

B. Lynch

C. Laurel

D. Precipice (Correct answer)

E. Unsure

92. Suffer [Multiple choice] *

A. Trend

B. Harrow

C. Add

D. Undergo (Correct answer)

E. Unsure

93. Assemble [Multiple choice] *

A. Neuro

B. Instigate

C. Aggregate (Correct answer)

D. Shock

E. Unsure

94. Reinforce [Multiple choice] *

A. Standard

B. Strengthen (Correct answer)

C. Force

D. Hear

E. Unsure

95. Accompany [Multiple choice] *

A. Afford

B. Attend (Correct answer)

C. Textbook

D. Migrate

E. Unsure

96. Hardship [Multiple choice] *

A. Rent

B. Affliction (Correct answer)

C. Anecdote

D. Soup

E. Unsure

97. Spouse [Multiple choice] *

A. Embrace

B. Organization

C. Mateship (Correct answer)

D. Sponsor

E. Unsure

98. Bulletin [Multiple choice] *

A. Stomach

B. Spend

C. Recite

D. Post (Correct answer)

E. Unsure

99. Undergo [Multiple choice] *

A. Experience (Correct answer)

B. Funeral

C. Confuse

D. Retain

E. Unsure

100. Diploma [Multiple choice] *

A. Diplomat

B. Jest

C. Certification (Correct answer)

D. Dilemma

E. Unsure

The test is over. Thanks for your participation!

Appendix 2: Vocabulary Retest Among College Students-Based on CET-4

Is your English vocabulary being attrited after summer vacation? This retest is in accordance with the test conducted before the last summer vacation. Please complete this retest and thanks for your cooperation. Again, when you are encountered with words that you are not sure about, please choose option E (unsure) for the accuracy of the test.

In addition, the information you fill in is all in the strictest confidence, and will only be used for research and analysis. I am here to promise that I will protect your privacy and security, and will not reveal it to any third party.

Name： [Fill in the blank] *

(It should be the same as the previous information.)

____

Gender： [Fill in the blank] *

____

Major： [Fill in the blank] *

____

1. Temporary [Multiple choice] *

A. Transient (Correct answer)

B. Temperature

C. Contemporary

D. Awesome

E. Unsure

2. Initiative [Multiple choice] *

A. Instead

B. Proposal (Correct answer)

C. Individual

D. Inactive

E. Unsure

3. Utilize [Multiple choice] *

A. Insignificant

B. Benthamism

C. Avail (Correct answer)

D. Ill-looking

E. Unsure

4. Subsequent [Multiple choice] *

A. Consequent (Correct answer)

B. Devastating

C. Substituted

D. Conquered

E. Unsure

5. Amateur [Multiple choice] *

A. Frustrated

B. Nonprofessional (Correct answer)

C. Accumulation

D. Mellow

E. Unsure

6. Abandon [Multiple choice] *

A. Sufferance

B. Ability

C. Ample

D. Discard (Correct answer)

E. Unsure

7. Withstand [Multiple choice] *

A. Retreat

B. Resist (Correct answer)

C. Intelligence

D. Understand

E. Unsure

8. Inflation [Multiple choice] *

A. Influence

B. Expansion (Correct answer)

C. Notice

D. Information

E. Unsure

9. Justify [Multiple choice] *

A. Certify (Correct answer)

B. Right

C. Judge

D. Travel

E. Unsure

10. Grind [Multiple choice] *

A. Grief

B. Grasp

C. Fragmentize (Correct answer)

D. Giant

E. Unsure

11. Halt [Multiple choice] *

A. Passage

B. Half

C. Stop (Correct answer)

D. Loath/ hate

E. Unsure

12. Seminar [Multiple choice] *

A. Service

B. Severe

C. Seminary

D. Symposium (Correct answer)

E. Unsure

13. Synthetic [Multiple choice] *

A. Artificial (Correct answer)

B. System

C. Symbol

D. Static

E. Unsure

14. Collapse [Multiple choice] *

A. Collude

B. Fall (Correct answer)

C. Collate

D. Dismantle

E. Unsure

15. Episode [Multiple choice] *

A. Expect

B. Express

C. Exercise

D. Series (Correct answer)

E. Unsure

16. Moisture [Multiple choice] *

A. Wetness (Correct answer)

B. Muzzle

C. Mystery

D. Instance

E. Unsure

17. Humble [Multiple choice] *

A. Unassuming (Correct answer)

B. Humorous

C. Hankering

D. Charitable

E. Unsure

18. Nuisance [Multiple choice] *

A. Numerous

B. Nature

C. Annoyance (Correct answer)

D. Nurse

E. Unsure

19. Stripe [Multiple choice] *

A. Naval

B. Strip

C. Variety (Correct answer)

D. Strike

E. Unsure

20. Fatigue [Multiple choice] *

A. Tiredness (Correct answer)

B. Fate

C. Coddle

D. Fair

E. Unsure

21. Accelerate [Multiple choice] *

A. Hasten (Correct answer)

B. Accent

C. Accept

D. Proximity

E. Unsure

22. Frustrate [Multiple choice] *

A. Member

B. Discouraging (Correct answer)

C. Melt

D. Fruit

E. Unsure

23. Penetrate [Multiple choice] *

A. Penalty

B. Pierce (Correct answer)

C. Episode

D. Practice

E. Unsure

24. Rigid [Multiple choice] *

A. Ride

B. Ridiculous

C. Upsurge

D. Tough (Correct answer)

E. Unsure

25. Dump [Multiple choice] *

A. Dumpling

B. Dumb

C. During

D. Fling/ trash (Correct answer)

E. Unsure

26. Recruit [Multiple choice] *

A. Entertain

B. Recent

C. Register (Correct answer)

D. Criticize

E. Unsure

27. Realm [Multiple choice] *

A. Domain (Correct answer)

B. Fact

C. Arrive

D. Dependence

E. Unsure

28. Saint [Multiple choice] *

A. Voyage

B. Sand

C. Deity (Correct answer)

D. Unitary

E. Unsure

29. Sensitive [Multiple choice] *

A. Sense

B. Delicate (Correct answer)

C. Adjudicative

D. Service

E. Unsure

30. Optical [Multiple choice] *

A. Choice

B. Optimistic

C. Opinion

D. Ocular (Correct answer)

E. Unsure

31. Demonstrate [Multiple choice] *

A. Democratic

B. Demand

C. Describe

D. Present (Correct answer)

E. Unsure

32. Accumulate [Multiple choice] *

A. Amass (Correct answer)

B. Accurate

C. Account

D. Actual

E. Unsure

33. Vibrate [Multiple choice] *

A. Village

B. Swing (Correct answer)

C. Vary

D. Vacation

E. Unsure

34. Tremendous [Multiple choice] *

A. Tendency

B. Treasure

C. Colossal (Correct answer)

D. Trace

E. Unsure

35. Sophisticated [Multiple choice] *

A. Spacious

B. Discreet

C. Complicated (Correct answer)

D. Sound

E. Unsure

36. Quotation [Multiple choice] *

A. Citation (Correct answer)

B. Quite

C. Qualification

D. Quiet

E. Unsure

37. Tropical [Multiple choice] *

A. Nutrient

B. Equatorial (Correct answer)

C. Storehouse

D. Malfunctioned

E. Unsure

38. Legislation [Multiple choice] *

A. Legality

B. Conceive

C. Insulate

D. Governance (Correct answer)

E. Unsure

39. Stale [Multiple choice] *

A. Suspend

B. Sleepy

C. State

D. Stinky (Correct answer)

E. Unsure

40. Barrier [Multiple choice] *

A. Obstruction (Correct answer)

B. Barrister

C. Barrel

D. Carrier

E. Unsure

41. Conscious [Multiple choice] *

A. Conscientious

B. Conservative

C. Prudent

D. Sentient (Correct answer)

E. Unsure

42. Flash [Multiple choice] *

A. Flesh

B. Flare

C. Flatter

D. Reflect (Correct answer)

E. Unsure

43. Breed [Multiple choice] *

A. Propagate (Correct answer)

B. Greed

C. Reed

D. Bread

E. Unsure

44. Intellectual [Multiple choice] *

A. Highbrow (Correct answer)

B. Intelligence

C. Institutional

D. Integrality

E. Unsure

45. Margin [Multiple choice] *

A. Marine

B. Edge (Correct answer)

C. Martyr

D. Swale

E. Unsure

46. Fulfill [Multiple choice] *

A. Accomplish (Correct answer)

B. Ideal

C. Irrespective

D. Improve

E. Unsure

47. Generous [Multiple choice] *

A. Moderate

B. Potent

C. Bounteous (Correct answer)

D. Innocent

E. Unsure

48. Insult [Multiple choice] *

A. Intrude

B. Explain

C. Instrument

D. Offend (Correct answer)

E. Unsure

49. Guarantee [Multiple choice] *

A. Hoggish

B. Associate

C. Pledge (Correct answer)

D. Instruct

E. Unsure

50. Indifferent [Multiple choice] *

A. Infantile

B. Unconcerned (Correct answer)

C. Inquire

D. Insist

E. Unsure

51. Opponent [Multiple choice] *

A. Inject

B. Springal

C. Hostile (Correct answer)

D. Superior

E. Unsure

52. Gown [Multiple choice] *

A. Frequency

B. Viewpoint

C. Frock (Correct answer)

D. Occasion

E. Unsure

53. Literary [Multiple choice] *

A. Library

B. Outlandish

C. Formal (Correct answer)

D. Announce

E. Unsure

54. Frontier [Multiple choice] *

A. Boundary (Correct answer)

B. Octagon

C. Imbed

D. Prescript

E. Unsure

55. Refine [Multiple choice] *

A. Appendage

B. Improve (Correct answer)

C. Previous

D. Question

E. Unsure

56. Combat [Multiple choice] *

A. Flame

B. Frog

C. Campaign (Correct answer)

D. Divide

E. Unsure

57. Capacity [Multiple choice] *

A. Spin

B. Disappointment

C. Exploitability

D. Competence (Correct answer)

E. Unsure

58. Starve [Multiple choice] *

A. Glossary

B. Infringe

C. Perish (Correct answer)

D. Corpse

E. Unsure

59. Cartoon [Multiple choice] *

A. Picture (Correct answer)

B. Ankle

C. Jargon

D. Freight

E. Unsure

60. Persist [Multiple choice] *

A. Restraint

B. Insist (Correct answer)

C. Elevator

D. Annals

E. Unsure

61. Fertile [Multiple choice] *

A. Fecund (Correct answer)

B. Futile

C. Hybrid

D. Fluent

E. Unsure

62. Freshman [Multiple choice] *

A. Immigration

B. Speech

C. Beginner (Correct answer)

D. Requirement

E. Unsure

63. Voyage [Multiple choice] *

A. Journey (Correct answer)

B. Snigger

C. Column

D. Impede

E. Unsure

64. Enhance [Multiple choice] *

A. Kettle

B. Wait

C. Disorient

D. Intensify (Correct answer)

E. Unsure

65. Exceed [Multiple choice] *

A. Dismantle

B. Practice

C. Surpass (Correct answer)

D. Opposition

E. Unsure

66. Perceive [Multiple choice] *

A. Contrive

B. Responsible

C. Detect (Correct answer)

D. Dandelion

E. Unsure

67. Cave [Multiple choice] *

A. Distort

B. Pit (Correct answer)

C. Settle

D. Transmission

E. Unsure

68. Handicap [Multiple choice] *

A. Obstacle (Correct answer)

B. Abundant

C. Requisite

D. Hole

E. Unsure

69. Jealous [Multiple choice] *

A. Envious (Correct answer)

B. Specialized

C. Vain

D. Unwilling

E. Unsure

70. Outlook [Multiple choice] *

A. Trauma

B. Purpose

C. Prospect (Correct answer)

D. Camara

E. Unsure

71. Dove [Multiple choice] *

A. Beach

B. Pigeon (Correct answer)

C. Self-esteem

D. Hailstone

E. Unsure

72. Flaw [Multiple choice] *

A. Call

B. Fault (Correct answer)

C. Dissolve

D. Breeze

E. Unsure

73. Privilege [Multiple choice] *

A. Peculiar (Correct answer)

B. Hummer

C. Mitigate

D. Accompanist

E. Unsure

74. Browse [Multiple choice] *

A. Garage

B. Aroma

C. Scan (Correct answer)

D. Resolve

E. Unsure

75. Addiction [Multiple choice] *

A. Admittance

B. Indulgence (Correct answer)

C. Committee

D. Avoidance

E. Unsure

76. Gather [Multiple choice] *

A. Evacuate

B. Collect (Correct answer)

C. Birth

D. Generate

E. Unsure

77. Riot [Multiple choice] *

A. Era

B. Invade

C. Violence (Correct answer)

D. Costa

E. Unsure

78. Bush [Multiple choice] *

A. Consent

B. Shrub (Correct answer)

C. Deficit

D. Meditate

E. Unsure

79. Wealth [Multiple choice] *

A. Property (Correct answer)

B. Health

C. Duck

D. Fineness

E. Unsure

80. Instruct [Multiple choice] *

A. Notebook

B. Train (Correct answer)

C. Argue

D. Demonstrate

E. Unsure

81. Dignity [Multiple choice] *

A. Jazz

B. Quantification

C. Sweater

D. Sanctity (Correct answer)

E. Unsure

82. Scan [Multiple choice] *

A. Skim (Correct answer)

B. Washroom

C. Reckon

D. Legend

E. Unsure

83. Cube [Multiple choice] *

A. Conviction

B. Solid (Correct answer)

C. Enigma

D. Longevity

E. Unsure

84. Supervise [Multiple choice] *

A. Oversee (Correct answer)

B. Doubt

C. Liquefaction

D. Nursing

E. Unsure

85. Remedy [Multiple choice] *

A. Queen

B. Therapy (Correct answer)

C. Trunk

D. Ready

E. Unsure

86. Intake [Multiple choice] *

A. Rose

B. Recommend

C. Entangle

D. Inhalation (Correct answer)

E. Unsure

87. Enthusiasm [Multiple choice] *

A. Increasement

B. Contract

C. Passion (Correct answer)

D. Implore

E. Unsure

88. Grasp [Multiple choice] *

A. Commute

B. Hinder

C. Catch (Correct answer)

D. Plantation

E. Unsure

89. Skeleton [Multiple choice] *

A. Bone (Correct answer)

B. Mercy

C. Broadcast

D. Habitant

E. Unsure

90. Cling [Multiple choice] *

A. Adhere (Correct answer)

B. Blame

C. Lurch

D. Example

E. Unsure

91. Cliff [Multiple choice] *

A. Bless

B. Lynch

C. Laurel

D. Precipice (Correct answer)

E. Unsure

92. Suffer [Multiple choice] *

A. Trend

B. Harrow

C. Add

D. Undergo (Correct answer)

E. Unsure

93. Assemble [Multiple choice] *

A. Neuro

B. Instigate

C. Aggregate (Correct answer)

D. Shock

E. Unsure

94. Reinforce [Multiple choice] *

A. Standard

B. Strengthen (Correct answer)

C. Force

D. Hear

E. Unsure

95. Accompany [Multiple choice] *

A. Afford

B. Attend (Correct answer)

C. Textbook

D. Migrate

E. Unsure

96. Hardship [Multiple choice] *

A. Rent

B. Affliction (Correct answer)

C. Anecdote

D. Soup

E. Unsure

97. Spouse [Multiple choice] *

A. Embrace

B. Organization

C. Mateship (Correct answer)

D. Sponsor

E. Unsure

98. Bulletin [Multiple choice] *

A. Stomach

B. Spend

C. Recite

D. Post (Correct answer)

E. Unsure

99. Undergo [Multiple choice] *

A. Experience (Correct answer)

B. Funeral

C. Confuse

D. Retain

E. Unsure

100. Diploma [Multiple choice] *

A. Diplomat

B. Jest

C. Certification (Correct answer)

D. Dilemma

E. Unsure

Appendix 3: Questionnaire

First of all, thank you for completing the test and retest of English vocabulary. Please spend 3-5 minutes to fill in the questionnaire below, the information contained in it is only used for academic research, and will not have any impact on your current study. Thank you from the bottom of my heart for your great support!

Then, please read the following statements carefully and choose the suitable option (s) according to your actual situation. Please note that the last four questions are multiple-choice questions.

101. Which level is your English at after your self-assessment? [Multiple choice] *

A. High-level group

B. Medium-level group

C. Low-level group

D. Hard to say

102. Have you ever been exposed to English during the summer vacation? [Multiple choice] *

A. Totally no (Please jump to the end of the questionnaire and submit your response sheet.)

B. Barely

C. Occasionally

D. Often

103. What is your frequency of English exposure during the summer vacation? [Multiple choice] *

A. Everyday

B. Five or Six days a week

C. Three or four days a week

D. One or two days a week

104. What is your average exposure time per day in English? [Multiple choice] *

A. About 0 min

B. About 0-30 min

C. About 30-60 min

D. About 60-120 min

E. > 120 min

105. Do you think there is a relationship between extracurricular exposure to English and your own English proficiency? [Multiple choice] *

A. No

B. Yes, but it’s not a strong relationship.

C. Yes, there is a strong relationship in them.

D. Unsure

106. What is your type of exposure? [You can choose more than one answer.] *

A. Listening

B. Speaking

C. Reading

D. Writing

E. Translation

107. How do you get in touch with English? [You can choose more than one answer.] *

A. Network media

B. Printed books

C. Digital newspapers and journals

D. Traveling abroad

E. Speaking to foreigners

F. Entertainments such as watching movie, listening to music, and playing games

G. Others： _________________

108. What's your reason for getting in touch with English in the summer vacation? [You can choose more than one answer.] *

A. Fulfilling the requirement of school

B. The need for examination

C. Self-improvement

D. Web push

E. Participating in entertainments like games

F. Others： _________________

109. Which of the following parts do you pay more attention to when you are exposed to English during the summer vacation? [You can choose more than one answer.] *

A. Lexeme

B. Pronunciation

C. Grammar

D. Syntax

E. Construction

F. Others: _________________

This is the end of the retest and the questionnaire. Thanks gain for your cooperation!
